# LONGITUDINAL TRENDS OF FAMILY CAREGIVERS’ FINANCIAL WELL-BEING AND SUPPORTIVE CARE SERVICE USE

**DOI:** 10.1093/geroni/igae098.0641

**Published:** 2024-12-31

**Authors:** Yin Liu, Kyungmin Kim, Melissa Ferguson, Niying Li, Josey Batura

**Affiliations:** Utah State University, Logan, Utah, United States; Seoul National University, Seoul, Republic of Korea; Utah State University, Logan, Utah, United States; University of Georgia, Athens, Georgia, United States; Utah State University, Providence, Utah, United States

## Abstract

The stress process model denotes financial well-being and caregiving support as two important resources for family caregivers. Financial well-being can be a critical factor in securing paid/formal care services at home and in community settings. When care-related out-of-pocket expenses are high, however, it can also become a secondary strain (i.e., financial burden) for caregivers. We conducted a longitudinal trend analysis, utilizing four waves of cross-sectional data collected over time from caregivers enrolled in the National Study of Caregiving study (i.e., family/unpaid caregivers of participants in the National Health and Aging Trends Study 1, 2, 11, and 12 rounds). The findings suggested that across waves, subjective financial difficulty was associated with income, and there were disparities in financial well-being (i.e., defined as subjective financial difficulty and income) by care recipients’ chronic health conditions as well as caregivers’ race/ethnic minority status and levels of education. Further, financial well-being (including subjective financial difficulty and income) was associated with using community-based care services such as respite programs (e.g., adult day care services) and paid help for personal care at home. Caregiving policy needs to consider caregiver financial well-being and financial disparities as factors affecting care service use.

